# Deltoid muscle shape analysis with magnetic resonance imaging in patients with chronic rotator cuff tears

**DOI:** 10.1186/1471-2474-14-247

**Published:** 2013-08-19

**Authors:** Dominik C Meyer, Stefan Rahm, Mazda Farshad, Georg Lajtai, Karl Wieser

**Affiliations:** 1 Orthopaedic Department, Balgrist University Hospital, University of Zurich, Rämistrasse 71, 8006, Zurich, Switzerland; 2 Orthopaedic Department, Private Hospital Maria Hilf, Radetzkystrasse 35 9020 Klagenfurt, Carinthia, Austria

**Keywords:** Rotator cuff tear, Pseudoparalysis, Deltoid muscle, Myotendinous retraction

## Abstract

**Background:**

It seems appropriate to assume, that for a full and strong global shoulder function a normally innervated and active deltoid muscle is indispensable. We set out to analyse the size and shape of the deltoid muscle on MR-arthrographies, and analyse its influence on shoulder function and its adaption (i.e. atrophy) for reduced shoulder function.

**Methods:**

The fatty infiltration (Goutallier stages), atrophy (tangent sign) and selective myotendinous retraction of the rotator cuff, as well as the thickness and the area of seven anatomically defined segments of the deltoid muscle were measured on MR-arthrographies and correlated with shoulder function (i.e. active abduction). Included were 116 patients, suffering of a rotator cuff tear with shoulder mobility ranging from pseudoparalysis to free mobility. Kolmogorov-Smirnov test was used to determine the distribution of the data before either Spearman or Pearson correlation and a multiple regression was applied to reveal the correlations.

**Results:**

Our developed method for measuring deltoid area and thickness showed to be reproducible with excellent interobserver correlations (r = 0.814–0.982).

The analysis of influencing factors on active abduction revealed a weak influence of the amount of SSP tendon (r = −0.25; p < 0.01) and muscle retraction (r = −0.27; p < 0.01) as well as the stage of fatty muscle infiltration (GFDI: r = −0.36; p < 0.01). Unexpectedly however, we were unable to detect a relation of the deltoid muscle shape with the degree of active glenohumeral abduction. Furthermore, long-standing rotator cuff tears did not appear to influence the deltoid shape, i.e. did not lead to muscle atrophy.

**Conclusions:**

Our data support that in chronic rotator cuff tears, there seems to be no disadvantage to exhausting conservative treatment and to delay implantation of reverse total shoulder arthroplasty, as the shape of deltoid muscle seems only to be influenced by natural aging, but to be independent of reduced shoulder motion.

## Background

Pseudoparalysis of the shoulder is a fascinating disorder. It encompasses the inability to elevate the arm to 90° in the presence of unrestricted passive range of glenohumeral motion and in the absence of any neurologic impairment [1]. Pseudoparalysis is associated with mostly large rotator cuff tears, however it is an inconsistent finding in those patients and it is unknown which factors might promote or prevent this condition.

Each muscle of the shoulder girdle contributes to shoulder movement according to its moment arm, line of action and the position of the glenohumeral joint [2]. In the rotator cuff deficient shoulder the most obvious influence on glenohumeral motion is the deltoid muscle. Its unimpaired innervation and function is a prerequisite when considering a reverse total shoulder arthroplasty (RTSA) as treatment of chronic rotator cuff deficient shoulders. When the deltoid contracts, the rotator cuff supports elevation/abduction and stabilizes the humeral head within the glenoid, creating a fulcrum on which the deltoid can lever to elevate the arm. In cases of massive tears of the rotator cuff, the stabilizing function of the rotator cuff is lost and the deltoid muscle contraction may lead to an anterior-superior humeral head translation and subluxation [3].

The deltoid muscle has been classically divided into three anatomical portions: Pars clavicularis, pars acromialis and pars spinalis. More recent literature has reported the presence of a fibrous frame, which is identifiable on magnetic resonance images and divides the deltoid muscle into seven segments based on the attachment of intramuscular tendons [4,5].

It seems appropriate to assume, that for a full and strong global shoulder function a normally innervated and active deltoid muscle is indispensable. A failure in muscle fiber recruitment, a poor muscle coordination or an imbalance of the different deltoid areas may consequently lead to an impaired shoulder function, especially in case of a massive rotator cuff tear. When analyzing anterosuperior subluxation of the humeral head in preliminary clinical observations, the posterior part of the deltoid appeared to be involved in lifting the humerus upward and, being wound around the posterior part of the humeral head, pushing the humerus anteriorly and superiorly, thus potentially contributing to anterosuperior escape.

We therefore hypothesized that a relative posterior hypertrophy or anterior atrophy of the deltoid might be associated with pseudoparalysis of the shoulder. Second, we hypothesized that vice versa, long-standing pseudoparalysis and disuse of the shoulder leads to progressive deltoid atrophy, possibly deleterious to later RTSA.

To test these hypotheses we set out to analyse the size and shape of the deltoid muscle on magnetic resonance imaging studies, to establish an according measurement algorithm and to analyse its influence on shoulder function and its adaption (i.e. atrophy) in functionally impaired shoulders.

## Methods

Our database of shoulder surgeries was retrospectively reviewed. Reviewed were patients, suffering from a rotator cuff tear, who were treated in our institution, either with direct arthroscopic repair (between 2008 and 2011), with a latissimus dorsi transfer, or implantation of a RTSA (both between 2005 and 2011). Inclusion criteria were: no antecedent shoulder surgery on the involved shoulder and MR-arthrography as well as Constant score (CS) [6] within 90 days prior to surgery. CS were completed prior to surgery from an orthopaedic resident and archived in our prospective shoulder database. All surgical reports were reviewed for exact description of tendons involved in the rotator cuff tear.

In our institution all patients underwent MR arthrography on a 1.5-T MRI unit (Symphony, Siemens) after injection of approximately 12 mL (range: 10–14 mL) of gadopendetatedimeglumine (Magnevist, Schering solution with a concentration of 2 mmol/L). The shoulder was placed in a dedicated receive-only shoulder coil with the arm in a neutral position and the thumb pointing vertically. MR-arthrography protocols included T1-weighted spin- echo images in the coronal oblique plane with fat saturation (792/20; section thickness, 3 mm; field of view, 160 × 160 mm; matrix size, 265 × 512), in the transverse plane (500/30; section thickness, 3 mm; field of view, 160 × 160 mm; matrix size, 256 × 512), and in the sagittal oblique plane (500/30; section thickness, 4 mm; field of view, 160 × 160 mm; matrix size, 256 × 512). T2-weighted fast spin-echo images (3,000/20; section thickness, 4 mm; field of view, 160 × 160 mm; matrix size, 256 × 512) and intermediate-weighted fast spin-echo images (2,350/20; section thickness, 4 mm; field of view, 160 × 160 mm; matrix size, 256 × 512) were obtained in the coronal oblique plane with fat saturation. Patients with technically comparable MR-arthrographies performed in other institutions were included into the study group.

MRI data was stored on a picture-archiving and communication system (PACS) workstation and the provider’s image analysis software was used for review and measurement of images.

The degree of fatty infiltration of all rotator cuff muscles were assessed on MRI according to Fuchs et al. [7], a grading system, which is based on the Goutallier classification [8], which has been established for computed tomographic scans. Grading was on the most lateral parasagittal T1 weighted image, on which the scapular spine was in contact with the scapular body. The stages identified were: Stage 0: normal muscle; stage 1: some fatty streaks; stage 2: manifest fatty infiltration but less fat than muscle; stage 3: as much fat as muscle; stage 4: more fat than muscle. An adapted global fatty degeneration index (GFDI) was calculated: Goutallier stage of supraspinatus (SSP) + infraspinatus (ISP) + subscapularis (SSC) muscle/3).

The appearance of (SSP) muscle atrophy was assessed using the tangent sign [9].

As previously described [10], the retraction of the SSP myotendinous unit was assessed on oblique coronal (perpendicular to the plane of the glenoid fossa) sections through the centre of the supraspinatus tendon. “Tear size” was measured as the distance from the lateral edge of the humeral articular cartilage to the lateral end of the tendon stump. The retraction of the tendon end (“tendon retraction”) and the myotendinous junction (“muscle retraction”) were measured as the distance from level of the glenoid to the free tendon end and the most lateral insertion of the muscle into the tendon, respectively. Transition areas with markedly increased signal intensity compared with normal cuff tissue were included in the tear size. Adjacent images were used to clarify the anatomic relationships if required. By subtracting the measured tendon retraction from the myotendinous retraction, the length of the SSP tendon stump was defined as the “tendon length”. For all measurements the articular sided junctions were used, as these showed an excellent interobserver reliability in a previous investigation [10].

**Figure 1:**
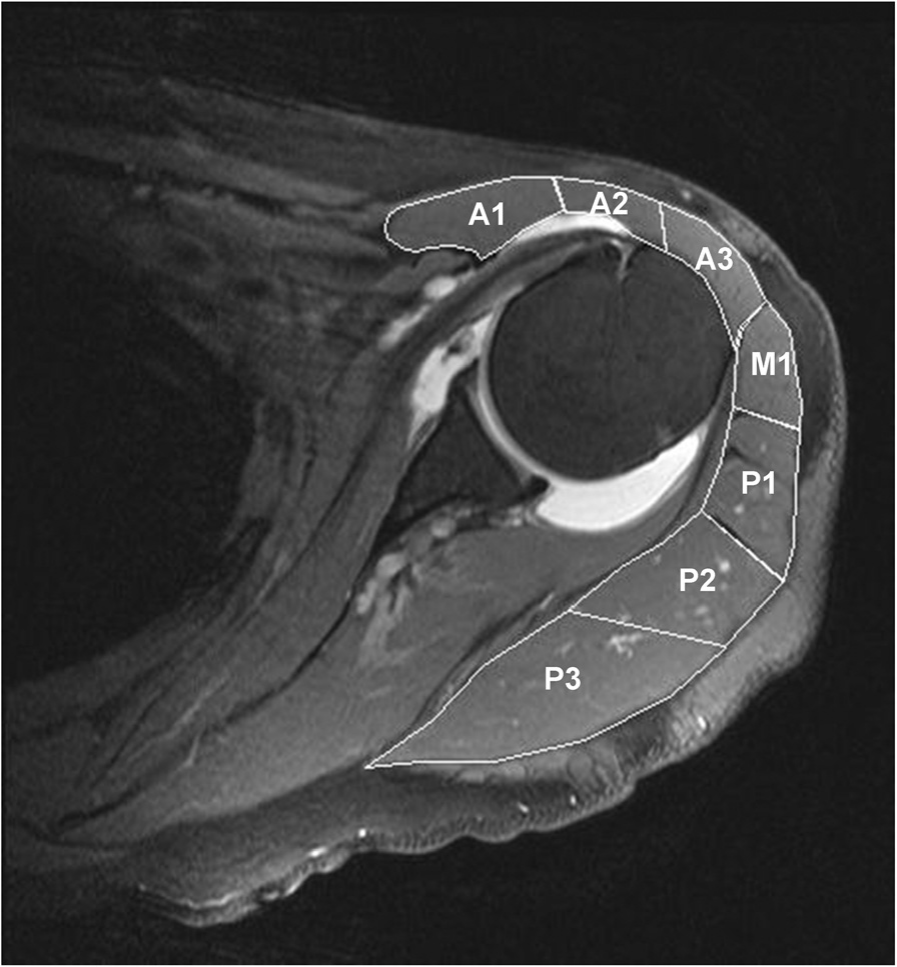
**Area of anatomic deltoid muscle segments.** On T2 weighted transverse MR images the area of seven anatomic defined segments of the deltoid muscle were measured exactly at the mid-glenoid level. These segments were identified following the visible tendons from their origin-points of the clavicle (A1), the anterior surface of the acromion (A2), the lateral aspect of the acromion (A3, M1 and P1) and the scapular spine (P2 and P3) respectively.

**Figure 2:**
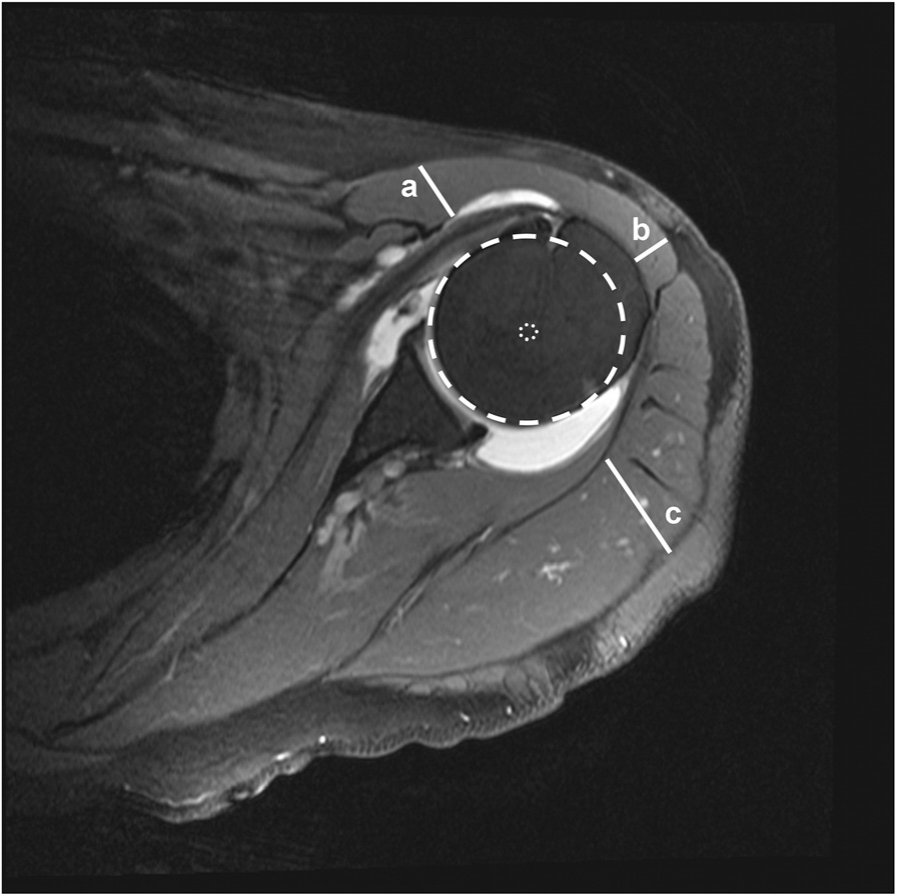
**Thickness of deltoid muscle.** The thickness of the ventral **(a)**, lateral **(b)** and dorsal **(c)** portion of the deltoid muscle was measured in designated directions from the center of humeral head (center of rotation) perpendicularly or parallel to the glenoid surface.

On T2 weighted transverse MR images the area of seven anatomically defined segments of the deltoid muscle were measured exactly at the mid-glenoid level (Figure 1). These segments were identified following the visible tendons from their origin-points of the clavicle (A1), the anterior surface of the acromion (A2), the lateral aspect of the acromion (A3, M1 and P1) and the scapular spine (P2 and P3) respectively [4,5]. Furthermore the thickness of the ventral, lateral and dorsal portion of the deltoid muscle was measured in designated directions from the center of humeral head (center of rotation) perpendicularly or parallel to the glenoid surface (Figure 2).

All included studies were randomly mixed and interpreted by independent readout of two orthopaedic surgeons (K.W. and S.R.) with 5 to 6 years of training in orthopaedic and particularly shoulder surgery.

Based on a general permit issued by the responsible state agency (“Schweizer eidgenössische Expertenkommission für das Berufsgeheimnis in der medizinischen Forschung”), the institutional review board of the Orthopaedic University Hospital Balgrist allows retrospective analysis of patient data relating to standard diagnostic or therapeutic procedures.

### Statistical analysis

Descriptive statistics was used to report basic measures. Values are given as mean and SD or range where appropriate. The statistical software PRISM (PRISM Version 5 for Mac, GraphPadInc) was used for correlation statistics under guidance of a medical professional trained in advanced statistics. Kolmogorov-Smirnov test was used to determine the distribution of the data before either Spearman (deltoid thickness/area; abduction) or Pearson (interreader) correlation was applied to the data to reveal the correlations. Logistic regression modeling (STATA software, StataCorp LP, Texas, USA) was used to account for potential interacting factor on relation of investigated parameters on shoulder abduction.

## Results

116 (45 women, 71 men) patients with a mean age of 60 years (range: 30–84) matched the inclusion criteria’s and were included in the analysis. In 82 patients the dominant arm (77 right, 5 left) and in 34 patients the non-dominant (3 right, 31 left) was involved.

The mean Constant score (CS) [6] was 49 (SD: 19; range: 12 – 94) points. The adapted CS was 60 (SD: 22; range: 17–100) %. The mean pain level was 8 (SD: 4; range: 0 – 15) points (15 points presents freedom of pain). And the mean abduction was 111 (SD: 48; range: 0 – 180) degrees. 70 patients were able to abduct more and 46 patients less than 90° respectively.

23 patients had a partial thickness tear of the SSP tendon, 10 without involvement of the remaining cuff, 8 with an additional SSC and 4 with an additional ISP lesion.

22 patients had a full thickness tear of SSP tendon, 32 of the SSP and ISP tendons, 20 of the SSP and SSC tendons, and 19 showed a massive tear with involvement of all three tendons.

The interreader correlations between the two observers are shown in Table 1. The analysis revealed an excellent correlation not only for the measurements of deltoid thickness (r = 0.847 – 0.921), but also for the measurements of areas and pars (r = 0.814 – 0.924), with the best correlation for the total deltoid area (r = 0.982).

**Table 1:** Interreader correlations

**Interreader correlations:**	**r-value**
**(Pearson)**	
Goutallier stages	0.91
SSP tendon retraction	0.96
SSP muscle retraction	0.94
Deltoid area pars spinalis	0.92
Deltoid area pars acromialis	0.81
Deltoid area pars clavicularis	0.89
Deltoid area total area	0.98
Deltoid thickness anterior	0.85
Deltoid thickness lateral	0.86
Deltoid thickness posterior	0.92

40 patients showed a positive tangent sign indicating a severe atrophy of the SSP muscle.

The Goutallier stages of the rotator cuff muscles are depicted in Table 2. The global fatty degeneration index (GFDI = Goutallier stage of SSP + ISP + SSC/3) showed a mean value of 1.4. Categorized in groups, the rounded index was 0 in 30, 1 in 30, 2 in 35, 3 in 19 and 4 in 2 patients.

**Table 2:** Goutallier stages of rotator cuff muscles

**Goutallier grading**	**Supraspinatus muscle**	**Infraspintus muscle**	**Subscapularis muscle**	GFDI*
**Stage 0 (n)**	29	41	33	30
**Stage 1 (n)**	21	19	41	30
**Stage 2 (n)**	28	20	21	35
**Stage 3 (n)**	19	24	15	19
**Stage 4 (n)**	19	12	6	2
**Mean Goutallier stage:**	1.7	1.4	1.1	1.4

*GFDI: global fatty degeneration index (=Goutallier stage of ((SSP + ISP + SSC)/3), rounded and classified into groups.

The mean tear size, measured from the footprint to the free tendon end was 18 (SD: 16; range: 0 – 52) mm. The free tendon end and the myotendinous junction were retracted to a mean of 23 (SD: 16; range: -13 – 49) mm and 6 (SD: 14; range: -25 – 36) mm to the level of the glenoid. The calculated tendon length (free tendon end to myotendinous junction) was 17 (SD: 5; range 4 – 30) mm.

The thickness and areas of the deltoid muscle are depicted in Table 3. The total area of the deltoid muscle measured 3896 (SD: 1317; range: 1566 – 7484) mm^2^. The anatomical pars spinalis showed with 1953 (SD: 728; range: 626 – 4028) mm^2^ the greatest area, followed by the pars acromialis (mean: 1242; SD: 421; range: 552 – 2609 mm^2^) and the pars clavicularis (mean: 702; SD: 306; range: 239 – 2133 mm^2^).

**Table 3:** Deltoid muscle thickness and area

	**Deltoid thickness (mm)**			**Deltoid area (mm**^**2**^**)**								**Deltoid pars (mm**^**2**^**)**		
	**Anterior**	**Lateral**	**Posterior**	**A1**	**A2**	**A3**	**M1**	**P1**	**P2**	**P3**	**Total**	Clavicularis*	Acromialis**	Spinalis***
**Mean:**	18	11	29	702	278	260	294	410	736	1217	3896	702	1242	1953
**Min:**	7	6	10	239	83	82	114	137	234	392	1566	239	552	626
**Max:**	45	22	52	2133	821	561	664	1001	1677	2714	7484	2133	2609	4028
***SD:***	*6*	*3*	*9*	*306*	*121*	*100*	*107*	*188*	*284*	*503*	*1317*	*306*	*421*	*728*

*pars clavicularis: A1; **pars acromialis: A2, A3, M1, P1; ***pars spinalis: P2, P3.

**Figure 3:**
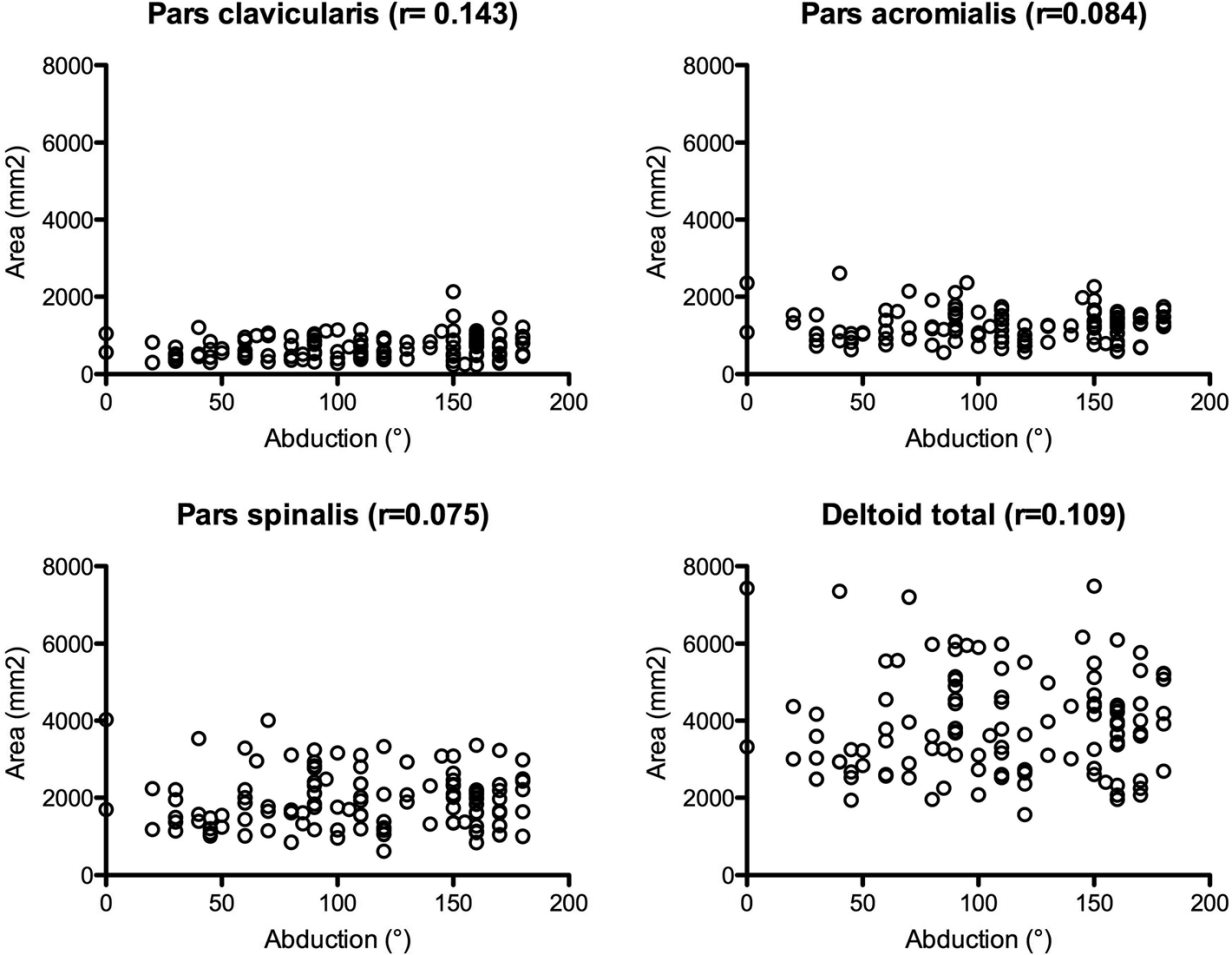
Correlation of the deltoid area.

The analysis of influencing factors on active abduction revealed a weak influence of the amount of SSP tendon (r = −0.25; p < 0.01) and muscle retraction (r = −0.27; p < 0.01) as well as the stage of fatty muscle infiltration (GFDI: r = −0.36; p < 0.01). Interestingly pain was found to be a not significant factor (r = 0.16; p > 0.05). Furthermore neither deltoid muscle thickness, nor deltoid area (r = 0.11; p > 0.05) showed a significant influence on active abduction excursion (Table 4; Figure 3).

**Table 4:** Correlation values of abduction

**Correlation of/to**	**Abduction**	
**(Spearman)**	**r-value**	***p-value***
**Deltoid thickness anterior**	0.14	*0.14*
**Deltoid thickness lateral**	−0.06	*0.5*
**Deltoid thickness posterior**	0.01	*0.94*
**Deltoid area pars clavicularis**	0.14	*0.13*
**Deltoid area pars acromialis**	0.08	*0.37*
**Deltoid area pars spinalis**	0.07	*0.42*
**Deltoid area total**	0.11	*0.25*
**SSP tendon retraction**	−0.25	*0.008*
**SSP Muscle retraction**	−0.27	*0.003*
**Goutallier SSP**	−0.35	*<0.0001*
**Goutallier ISP**	−0.29	*0.002*
**Goutallier SSC**	−0.28	*0.002*
GFDI*	−0.36	*<0.0001*
**SSP Atrohpy (Tangent sign)**	−0.27	*0.003*
CS** Pain	0.16	*0.08*

*GFDI: global fatty degeneration index (= Goutallier stage of (SSP + ISP + SSC)/3).

**Constant Score.

Not surprisingly did the deltoid area (r = −0.39; p < 0.01) and thickness (r = −0.31 - −0.41; p < 0.01)decrease with age. However rotator cuff tears did not show to have any influence on the deltoid muscle, as neither tendon (r = 0.021; p > 0.05) and muscle (r = 0.077; p > 0.05) retraction, nor the fatty infiltration (GFDI) (r = −0.054; p > 0.05), as marker of chronicity of the tear, could be identified as influencing factor on the area of the muscle (Table 5).

**Table 5:** Correlation values of deltoid thickness and area

**Correlation of/to**	**Deltoid thickness anterior**		**Deltoid thickness lateral**		**Deltoid thickness posterior**		**Total deltoid area**	
**(Spearman)**	**r-value**	***p-value***	**r-value**	***p-value***	**r-value**	***p-value***	**r-value**	***p-value***
**Age**	−0.41	*<0.0001*	−0.31	*0.001*	−0.35	*0.001*	−0.39	*<0.0001*
**SSP tendon retraction**	−0.05	*0.58*	0.06	*0.51*	0.03	*0.79*	0.02	*0.82*
**SSP muscle retraction**	0	*0.99*	0.12	*0.19*	0.07	*0.45*	0.08	*0.41*
GFDI*	−0.13	*0.14*	−0.03	*0.77*	−0.02	*0.85*	−0.05	*0.56*

*GFDI: global fatty degeneration index (= Goutallier stage of (SSP + ISP + SSC)/3).

## Discussion

The deltoid is an important shoulder muscle, contributing to shoulder flexion, abduction and extension. With deltoid contraction, there is a cranial directed force on the humerus, which is countered by the shoulder-stabilizing muscles such as the rotator cuff, creating a dynamic fulcrum. Further, the deltoid is deflected by the humeral head and compresses the humeral head distally and against the glenoid [11]. However the deltoid has a far greater diameter posteriorly than anteriorly (Figure 2). Therefore, we hypothesized that there is also an anterior-directed force on the humeral head, which – in case of rotator cuff deficiency - may create an antero-superior shift of the humeral head resulting in dynamic anterosuperior instability and associated shoulder pseudoparalysis. Further, we hypothesized that with long-standing rotator cuff tears the deltoid will lose volume and further weaken shoulder function.

Against our expectations however, both our hypotheses could not be confirmed. Though we could establish a very repeatable measurement method, we were unable to detect any influence of the deltoid shape on the amount of active glenohumeral abduction. Furthermore, longstanding rotator cuff tears with massive retraction and fatty infiltration of the cuff muscles did not turn out to be associated with a loss of deltoid volume, i.e. did not lead to muscle atrophy.

The deltoid muscle was classically divided into three parts: the clavicular or anterior, the acromial or medial, and the spinal or posterior portions. More recent anatomic literature divided the deltoid muscle into seven segments based on the attachment of intramuscular tendons [4,5]. The multipennate organization of these intramuscular tendons favors strength instead of excursion of the deltoid muscle [12] and has been found to be identifiable on magnetic resonance images [4]. Recent fludeoxyglucose positron emission tomography (FDG-PET) studies indicated that the deltoid muscle worked as segments during the arm elevation, which corresponded well to the anatomically defined seven muscular segments with the intramuscular tendons [5]. This study is to our knowledge the first to assess the deltoid shape in the context of rotator cuff tears and shoulder function.

Due to its retrospective design our chosen model analyzing preoperatively collected data might have some potential drawbacks, like interaction of different cuff tear patterns or severe pain. The amount of retraction was measured in a standardized fashion through the center of tendon. However, it might be possible that a larger part of the tear was located at another area in the rotator cuff. Therefore our results might underestimate the influence of the cuff tear extension and retraction on shoulder abduction. Furthermore the analysis and correlations of the deltoid area and function are limited to absolute values and were not normalized to patients’ height, weight or body mass index and does therefore not take account of the size of each subject. Additionally, we used a logistic regression model to find associative factors to shoulder abduction and to account for potential interactions, such as pain and tendon tear pattern. The amount of SSP tendon and muscle retraction, as well as the degree of fatty infiltration and degeneration of all muscle of the rotator cuff were identified as correlating factors.

In case of tendon tears, disuse, denervation, cachexia or myodystrophic disease muscular atrophy is a common and unavoidable consequence. The pathophysiologic mechanisms and the histological and biochemical changes differ greatly between these different etiologies, however fatty infiltration and the consequent decrease of muscle cross sectional area results in declined muscle strength, elasticity and range of joint motion [13-15].

We could identify increasing age to negatively influence the deltoid shape, with a decreased area and thickness of the deltoid muscle in elderly patients. Still, our second assumption, that in case of a chronic rotator cuff tear with pseudoparalysis and therefore decreased use of the shoulder, atrophy of the deltoid muscle may occur, had to be rejected as well. This is an interesting and unexpected finding in two aspects: First, the mostly isometric contraction of the deltoid in pseudoparalytic shoulders seems to be sufficient to prevent measurable deltoid atrophy. This supports the use of isometric strengthening exercises in rehabilitation. Second, as a reasonably preserved function and structure of the deltoid muscle is a prerequisite for reverse total shoulder arthroplasty, the data presented in this study supports the possibility to exhaust conservative treatment options as there is no need to rush RTSA in order to preserve the deltoid muscle.

## Conclusions

Measurement of the deltoid cross sectional area and thickness is reproducible on MR arthrographies with detectable intramuscular tendons, dividing functional muscle segments. However, in this radiographic analysis, deltoid muscle shape could neither be identified as a determining factor for shoulder function in the pseudoparalytic shoulder, nor could we detect severe atrophy of the deltoid muscle as a result of long standing rotator cuff tears with pseudoparalysis. We conclude that also with advanced pseudoparalysis there seems to be little risk for deleterious deltoid atrophy, which supports the use of RTSA also for patients with long-standing loss of shoulder function.

## Consent

In accordance with this applicable state law, the hospital’s institutional review board has issued a general permit for retrospective review of image data based on the hospital’s policy protecting patient privacy, which includes the patiens’ right to reject the use of their image data for scientific purposes.

## Abbreviations

CS: Constant score; SSP: Supraspinatus; ISP: Infraspinatus; SSC: Subscapularis; TM: Teres minor; GFDI: Global fatty degeneration index; RTSA: Reverse total shoulder arhtroplasty.

## Competing interests

None of the authors did receive anything of value from or own stock in a commercial company related to the subject of this article.

## Authors’ contributions

All authors have made substantial contributions to this study. DCM was involved in conception and design and interpretation of data and revising the manuscript. SR and MF were involved in the acquisition evaluation and statistical analysis of data. GL was involved in conception and design and revising the manuscript critically for important intellectual content. KW was involved in conception and design and interpretation of data and drafting the manuscript. All authors read and approved the final manuscript.

## Pre-publication history

The pre-publication history for this paper can be accessed here:

http://www.biomedcentral.com/1471-2474/14/247/prepub
